# Supplementary material for: Identification and functional analysis of non-coding regulatory small RNA FenSr3 in Bacillus amyloliquefaciens LPB-18
Source: PeerJ. 2023 May 15;11:e15236. doi: 10.7717/peerj.15236 (PMC10194069; doi:10.7717/peerj.15236)
Supplement: Supplemental Information 4 [file peerj-11-15236-s004.zip › KO/CK-vs-T1_map/map05203.html]

KEGG PATHWAY: Viral carcinogenesis - Reference pathway


|  |  |
| --- | --- |
| **Viral carcinogenesis - Reference pathway** |  |

[
Pathway menu
| Organism menu
| Pathway entry
| Show description
| User data mapping
]

There is a strong association between viruses and the development of human malignancies. We now know that at least six human viruses, Epstein-Barr virus (EBV), hepatitis B virus (HBV), hepatitis C virus (HCV), human papilloma virus (HPV), human T-cell lymphotropic virus (HTLV-1) and Kaposi's associated sarcoma virus (KSHV) contribute to 10-15% of the cancers worldwide. Via expression of many potent oncoproteins, these tumor viruses promote an aberrant cell-proliferation via modulating cellular cell-signaling pathways and escape from cellular defense system such as blocking apoptosis. Human tumor virus oncoproteins can also disrupt pathways that are necessary for the maintenance of the integrity of host cellular genome. Viruses that encode such activities can contribute to initiation as well as progression of human cancers.

|  |  |  |
| --- | --- | --- |
| Reference pathway | 184% 150% 122% 100% 82% 67% 55% | 图片下载 |
